# Cost effectiveness and resource allocation of *Plasmodium falciparum* malaria control in Myanmar: a modelling analysis of bed nets and community health workers

**DOI:** 10.1186/s12936-015-0886-x

**Published:** 2015-09-29

**Authors:** Tom L. Drake, Shwe Sin Kyaw, Myat Phone Kyaw, Frank M. Smithuis, Nicholas P. J. Day, Lisa J. White, Yoel Lubell

**Affiliations:** Mahidol-Oxford Tropical Medicine Research Unit, 420/6 Rajvithi Rd, Bangkok, 10400 Thailand; Nuffield Department of Medicine, University of Oxford, Oxford, UK; Department of Medical Research, Ministry of Health, Yangon, Myanmar; Medical Action Myanmar, Yangon, Myanmar

**Keywords:** Malaria, Economic, Cost, Cost effectiveness, Policy, Resource allocation

## Abstract

**Background:**

Funding for malaria control and elimination in Myanmar has increased markedly in recent years. While there are various malaria control tools currently available, two interventions receive the majority of malaria control funding in Myanmar: (1) insecticide-treated bed nets and (2) early diagnosis and treatment through malaria community health workers. This study aims to provide practical recommendations on how to maximize impact from investment in these interventions.

**Methods:**

A simple decision tree is used to model intervention costs and effects in terms of years of life lost. The evaluation is from the perspective of the service provider and costs and effects are calculated in line with standard methodology. Sensitivity and scenario analysis are undertaken to identify key drivers of cost effectiveness. Standard cost effectiveness analysis is then extended via a spatially explicit resource allocation model.

**Findings:**

Community health workers have the potential for high impact on malaria, particularly where there are few alternatives to access malaria treatment, but are relatively costly. Insecticide-treated bed nets are comparatively inexpensive and modestly effective in Myanmar, representing a low risk but modest return intervention. Unlike some healthcare interventions, bed nets and community health workers are not mutually exclusive nor are they necessarily at their most efficient when universally applied. Modelled resource allocation scenarios highlight that in this case there is no “one size fits all” cost effectiveness result. Health gains will be maximized by effective targeting of both interventions.

**Electronic supplementary material:**

The online version of this article (doi:10.1186/s12936-015-0886-x) contains supplementary material, which is available to authorized users.

## Background

Malaria in Myanmar is important not only because of the health burden to the country’s own population, but because of the emergence of artemisinin resistant *Plasmodium falciparum* parasites in the region [1–3]. The burden of malaria in Myanmar is spatially heterogeneous and seasonal. An estimated 37 % of the population live in areas broadly considered at high risk of malaria (>1 case per 1000 population) and a further 23 % live in areas of low malaria risk (0–1 cases per 1000 population) [4]. Funds for malaria control and elimination in Myanmar have surged in recent years, including the Myanmar specific Three Millennium Development Goal (3MDG) fund and the Global Fund’s Regional Artemisinin Initiative; a US$ 100 million fund of which US$ 40 million has been allocated to Myanmar. The financial resources available to Myanmar at this time are both unprecedented in size and potentially time limited. It is critical, therefore, that these resources are allocated efficiently; maximizing impact and improving financially sustainability.

While there are various malaria control tools currently available, two interventions receive the majority of malaria control funding in Myanmar (1) insecticide-treated bed nets (ITN), including long-lasting insecticide-treated nets and (2) early diagnosis and treatment through malaria community health workers (CHW). ITN are most effective against mosquitoes which are nocturnal, endophagic blood feeders whereas most species commonly found in Myanmar tend toward crepuscular and exophagic biting [5–7]. The evidence base for the cost effectiveness of ITN against malaria spread by the former type of mosquito is strong [8] and previous modelling analysis found that while changes in mosquito biting behaviour could reduce effectiveness, nevertheless ITN could remain a cost effective intervention [9]. Malaria CHW costs have been estimated in Cambodia [10], Nigeria [11] and across sub-Saharan Africa [12].

The malaria policy discourse in Myanmar is frequently framed as a choice between prioritizing universal coverage of either ITN or CHW. While ITN and CHW can be thought of as competing for limited resources they are not mutually exclusive interventions and are in many senses complimentary. It is also the case however that funding is not available for universal access to both interventions, nor has it been demonstrated that such scale-up would be an efficient use of scarce resources in all settings. The factors which determine the costs and effects of both interventions will vary across the country, and context is important in understanding cost effectiveness. This study evaluates the costs and effects of these key malaria control interventions in Myanmar with an emphasis on sensitivity and scenario analysis rather than a generalized cost effectiveness result. Furthermore, targeted allocation of these resources is illustrated by an allocation model for a region of Myanmar.

## Methods

### Costing

Financial costs are included from the perspective of the National Malaria Control Programme or other malaria intervention funders. In this analysis ITN distribution is assumed to be conducted though a dedicated distribution campaign. ITN cost is comprised of procurement cost (*c*_*p*_), direct distribution costs (*c*_*d*_) and programme management (*c*_*m*_). Cost data were obtained from Three Millennium Development Goal (3MDG), a funding organization in Myanmar, with crosschecking of components against private sector quotations. A distribution of two nets per household is assumed with 10 % wastage (*w*) and a mean household size of 5.2 people. The primary time horizon is one year and as such the per person ITN cost is annualized according to the lifespan of the net (*l*), assumed to be three years, using a discount rate of 5 % (*r*) [13].

1 $$c_{ITN} = \frac{{(c_{p} + c_{d} + c_{m} )(1 + w)}}{{r^{ - 1} \left( {1 - \left( {1 + r} \right)^{ - l} } \right)}}$$

CHW costs are derived from separate detailed cost analysis currently under review. To briefly summarize, CHW costs are estimated using an ingredients based micro costing of six cost centres: patient services; training; monitoring and supervision, programme management; incentives and overheads. For this cost effectiveness analysis the cost of treatment (*c*_*ACT*_) is separated from the remaining CHW cost per person covered (*c*_*CHW*_). In addition to intervention costs, diagnosis and treatment direct costs for malaria cases treated by the basic health system are included (*c*_*ACT*_).

### Model

CHW are an extension of the health system and therefore marginal utility will depend on locally specific access to treatment. The model must define a common metric to quantify the effects of ITN and CHW. The model calculates the number of years of life lost (YLL), a widely used metric for health impact, through treatment of cases or cases directly averted by bed nets. In this case YLL are likely to be similar to disability adjusted life years as the contribution of morbidity will be negligible compared with mortality. The model was developed in both R (version 3.1.2) and TreeAge (TreeAge Pro 2014, USA).

**Fig. 1:**
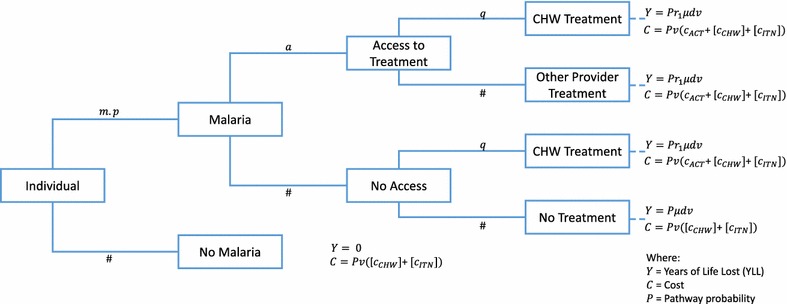
Probability tree model of cost and impact for malaria community health workers and bed nets

The probability tree (Fig. 1) traces an individual through a chronological series of event possibilities beginning with an annual probability of contracting malaria (*m*) which is adjusted by the protective effect of ITN (*p*), if applicable. Individuals with malaria have a probability they will receive treatment from a provider other than a CHW (*a*). If a CHW is available in the village there is a probability (*q*) that a malaria case will seek treatment from the CHW, from both those who would have received treatment elsewhere and from those who would not have received any treatment. Each case of malaria has a probability of death in absence of treatment (*μ*) and a mean number of YLLs lost per death (*d*). Treatment is assumed to be with an ACT. The direct reduction in mortality is assumed to be the same for ACT (*r*_1_). The terminal payoffs are scaled by population (*v*) and calculate the net cost and net effects for each intervention arm for one village (or one township when applied in the resource allocation model, see below). Parameter values can be found in Table 1. For the purpose of this model only one provider is attended per person, individuals may seek treatment at a CHW instead of their previous provider. This is intended to reflect the greater marginal utility is areas with poor access to treatment, even when uptake at the CHW is equal.

**Table 1:** Parameter list and values for decision tree models

	Model parameter	Symbol	Default value	Lower estimate	Upper estimate	Source
Setting	Baseline access to treatment (% of cases receiving ACT)	*a*	30 %	1 %	95 %	2011 MARC survey indicates low availability, but recently survey by PSI indicates a substantial increase
	Cost of treatment	*c*_*ACT*_	$3	1	10	Wholesale price of diagnosis and treatment, consumables only.3MDG
	Proportion of malaria cases that die in absence of treatment	*μ*	1 %	0.1 %	10 %	Expert opinion [14]
	Probability of getting malaria	*m*	5 %	0.1 %	30 %	Probability of malaria is highly variable but changes do not affect comparative analysis between intervention options
	Probability that a person with malaria uses a CHW (where available)	*q*	30 %	1 %	95 %	Community survey by Department of Medical Research in Myanmar finds 19 % of surveyed first seek treatment at CHW (unpublished). Community survey in Cambodia finds low utilisation of CHW in villages with a CHW (Yeung et al. unpublished)
	Mean number of disability adjusted life years lost per death	*d*	30	15	45	Assumed based on life expectancy of 65 years and knowing that most malaria deaths in Myanmar are adults
	Village population	*v*	500	–	–	Village size is based on unpublished unicef data. At the time of the study the village level census data was unavailable
Intervention	Annual cost of ITN per person	*c*_*ITN*_	$0.70	$0.50	$1.5	Estimated
	Annual cost of CHW per person	*c*_*CHW*_	$2	$1.10	$4.50	Kyaw et al. under review
	ITN protective efficacy	*p*	30 %	0 %	50 %	[5, 9, 15, 16]
	Reduction in mortality after treatment with ACT or ACT + PQ	*r*_1_	90 %	50 %	99 %	Expert opinion

The model was developed as the simplest structure that incorporates the key relevant data and provides the desired output metrics of cost and years of life lost. The advantages of a simple model are ease of communication to end users, speed of development and flexibility of application.

### Analysis

Bed nets and community health workers are not universally applied interventions and a general estimate of intervention costs and effects misses important variation, particularly with respect to the sometimes extreme remoteness of different populations in Myanmar. Instead, intervention cost effectiveness is calculated in four illustrative accessibility or remoteness scenarios, whereby more remote settings are characterized by increased cost of programme delivery, increased CHW uptake and decreased baseline access to treatment (Table 2). Data are not available to support specific parameterizations for these assumption but the direction of trends are intuitive and supported by policy makers at the national malarial control programme and programme managers at an affiliated non-governmental organization, Medical Action Myanmar. In addition to the scenario analysis, univariate sensitivity analysis is undertaken to identify key determinants of intervention cost effectiveness. Probabilistic Sensitivity Analysis (PSA) can be found in the supporting documentation (Additional File 1). Quantified and non-quantified costs and consequences are summarized in Table 3 to aid interpretation and to highlight potentially important factors which are not included in the quantitative analysis, as recommended for economic evaluations of public health interventions by Weatherly and colleagues [17].

**Table 2:** Parameter values for four remoteness scenarios

Parameter	Symbol	Remoteness scenario			
		Easily accessible	Accessible	Difficult to access	Very difficult to access
Annual cost of VHW per person	*c*_*CHW*_	1.10	2.00	3.20	4.50
Annual cost of LLIN per person	*c*_*ITN*_	0.5	0.70	1.2	1.5
Probability that a person with malaria utilises a VHW (where available)	*q*	0.15	0.3	0.45	0.6
Baseline access to treatment (% of cases receiving ACT)	*a*	0.5	0.3	0.15	0

**Table 3:** Cost-consequence summary of insecticide treated nets and malaria community health workers in Myanmar

	ITN	CHW
Direct costs	One off purchase and distribution costs are annualised over the lifespan of the net Annual equivalent cost per village in modelled scenarios: US$ 240–750	Annual costs include: training, patient services, monitoring and supervision, programme management and CHW remuneration or incentives. Annual cost range in modelled scenarios (excluding variable drug costs): US$ 560–2300 Although the effective cost for malaria funds could be reduced through cost sharing
Direct consequences	Modest impact on malaria disease in Myanmar due to crepuscular and exophagic biting	High impact on malaria disease if there is good utilisation of the CHW by people who have malaria
Indirect consequences	Modest impact on malaria transmission in Myanmar due to crepuscular and exophagic biting	High impact on malaria transmission if there is good utilisation of the CHW by people who have malaria
	Direct effects of ITN result in use of fewer diagnostics and treatment and therefore save some costs (included in analysis)	CHW can be used to provide other health services, feedback valuable information on malaria burden, provide information and educational messages to the community (not included in analysis)

Cost effectiveness ratios are calculated for each intervention against a common null comparator or “no additional intervention” baseline, which includes the number of YLLs expected in absence of intervention and the cost of treatment for patients who receive it. The marginal benefit of each in the presence of the other is not equal to the marginal benefit of each in isolation. A CHW in a village with good bed net coverage has lower impact than in the same village without bed net coverage because there are fewer cases to treat, and vice versa. For this reason the combined intervention arm is included explicitly as a model output rather than as a sum of separate interventions. Estimates are per year and reflect a village of 500 people with 25 malaria cases per year in absence of interventions.

### Resource allocation

An extension to standard cost effectiveness analysis, the second stage of this study applies a spatially explicit resource allocation model for a given budget. The model is applied to the Tier 1 or ‘MARC’ region of Myanmar, an area in the east of Myanmar identified as a priority area for malaria control. There are 52 townships in Tier 1 to which a fixed budget of US$ 10 million is allocated. Township specific data on population is from the 2014 census [18] and malaria incidence is based on routine health system surveillance records, currently managed by WHO Myanmar on behalf of the Ministry of Health (2013, unpublished). The malaria surveillance system in Myanmar is undergoing systemic improvements and data capture is not complete. All other parameter values are as reported in Table 1.

The allocation model uses the decision tree in Fig. 1 to calculate cost effectiveness ratios for all intervention options for each geographic patch, in this case a township. Once all scenario cost effectiveness ratios are calculated the model allocates the available budget starting with the most cost effective intervention. As the budget is allocated, the most cost effective intervention in a particular township may be replaced by a less cost effective, but more effective intervention. Dominated intervention scenarios, those where any increase in effect can be achieved by a more cost effective alternative, are excluded. The allocation process ceases when the remaining budget is less than the marginal cost of the next most cost effective intervention. It is worth noting that the optimal allocation of resources is not identified through sequential iteration and improvement of budget allocation options since the cost effectiveness ratios provide sufficient information to identify the allocation result directly. This is more accurate and computationally efficient than identification of a distribution of resources through iterative optimization or “brute-force” calculation of all or a large number of possible distribution scenarios. The resource allocation analysis is repeated to examine the impact of variations in bed net protective effectiveness, CHW uptake and cost sharing for integrated CHW programmes.

## Results

**Fig. 2:**
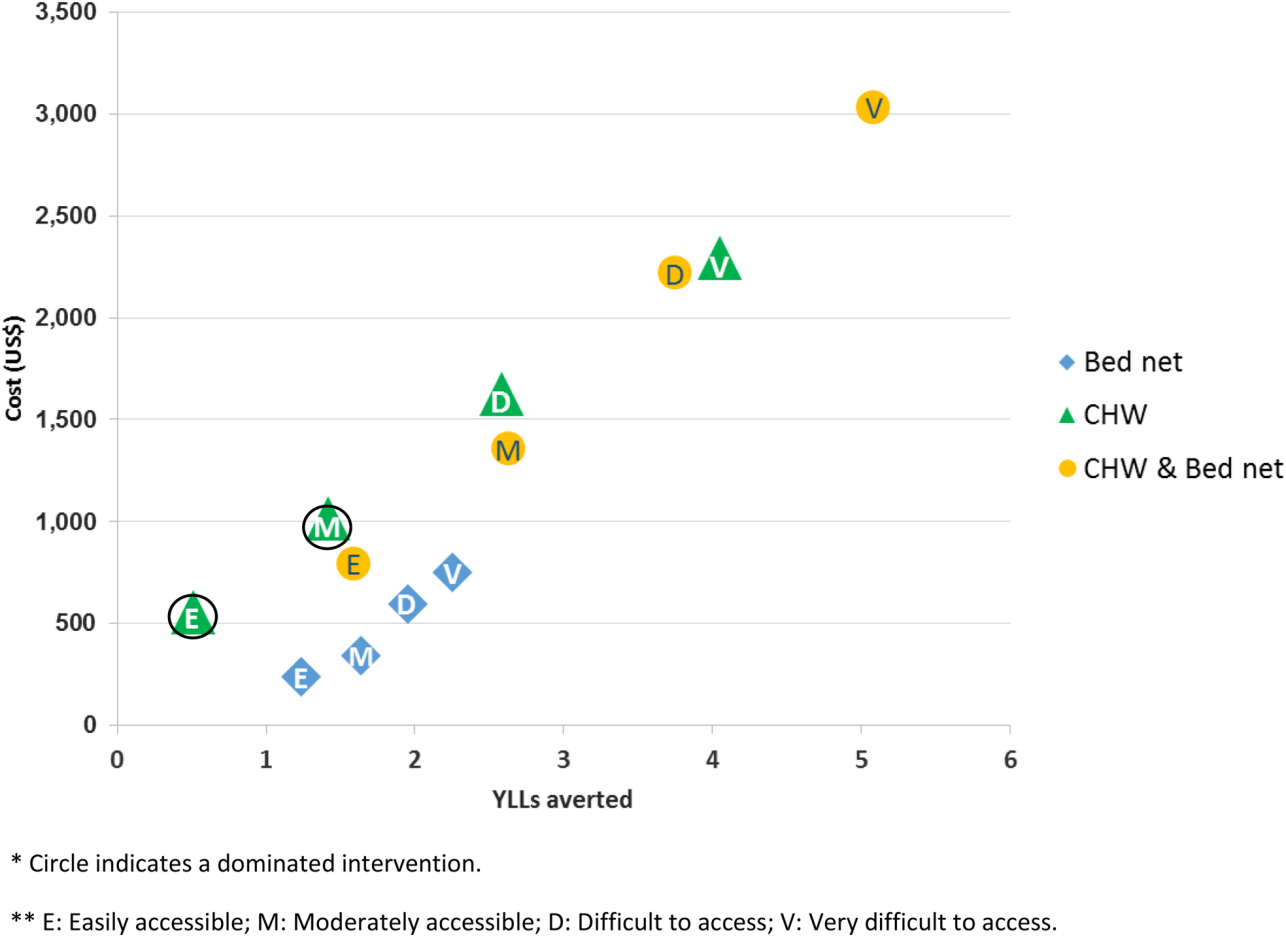
Costs and effects of malaria control in different accessibility scenarios. *Circle* indicates a dominated intervention. *E* easily accessible, *M* moderately accessible, *D* difficult to access, *V* very difficult to access

The cost effectiveness of malaria control in Myanmar is context dependent. CHW have greater potential effects, particularly in more remote settings, but are also more costly. In the scenario analysis, easily accessible village setting CHW avert 0.51 YLLs per year at a cost of US$ 556 (US$ 1089 per YLL averted). This rises in the very difficult to reach villages to 4.05 YLLs averted at a cost of US$ 2295 (US$ 567 per YLL averted), a higher cost but a more cost effective use of CHWs. Bed nets were consistently less costly and a modestly effective intervention. In the easily accessible village setting bed nets avert 1.24 YLLs at a cost of US$ 238 (US$ 193 per YLL averted), rising to 2.25YLL averted for US$ 750 (US$ 333 per YLL averted). In the very difficult to access village setting, a combination of both bed nets and CHW gives the greatest impact of 5.08 YLLs averted for a cost of US$ 3031 (US$ 597 per YLL averted). The above results are summarized in Table 4 and Fig. 2 and assume that CHW only provide malaria services (this assumption is relaxed in the resource allocation analysis).

**Table 4:** Costs and effects of malaria interventions in four remoteness scenarios

	Remoteness			
	Easily accessible	Accessible	Difficult to access	Very difficult to access
ITN				
Cost (US$)	238	343	596	750
Effect (YLLs averted)	1.24	1.64	1.95	2.25
CER*	193	209	306	333
CHW				
Cost (US$)	556	1016	1629	2295
Effect (YLLs averted)	0.51	1.42	2.58	4.05
CER*	1089	715	631	567
ICER**	Abs dominated	Abs dominated	Ext dominated	Ext dominated
CHW and ITN				
Cost (US$)	792	1354	2216	3031
Effect (YLLs averted)	1.59	2.63	3.75	5.08
CER*	499	515	591	597
ICER**	1583	1021	503	715

* CER here compares costs and effects of an intervention compared with no intervention

** ICER compares costs and effects of an intervention compared with the next most effective undominated option

### Sensitivity analysis

**Fig. 3:**
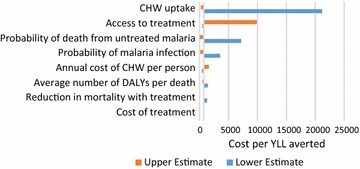
Change in CHW cost effectiveness: univariate sensitivity analysis of all relevant parameters

**Fig. 4:**
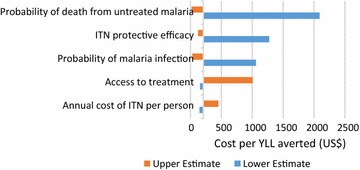
Change in bed net cost effectiveness: univariate sensitivity analysis of all relevant parameters

Univariate sensitivity analysis was conducted for the cost effectiveness of CHW (Fig. 3) and bed nets (Fig. 4) using the wide uncertainty ranges in Table 1. The key determinants of cost effectiveness for CHW are baseline access to treatment with an ACT and the likelihood that a person with malaria seeks treatment from the CHW. In reality these two factors may be related; low baseline access to treatment might be expected to increase treatment seeking at a CHW. Univariate sensitivity analysis treats these values as independent. The key determinants of bed net cost effectiveness are the untreated malaria mortality risk and the protective effect of the net. Changes in malaria incidence and mortality affect the magnitude of effects substantially but proportionally for all intervention options, and therefore do not affect intervention comparison.

### Resource allocation

**Fig. 5:**
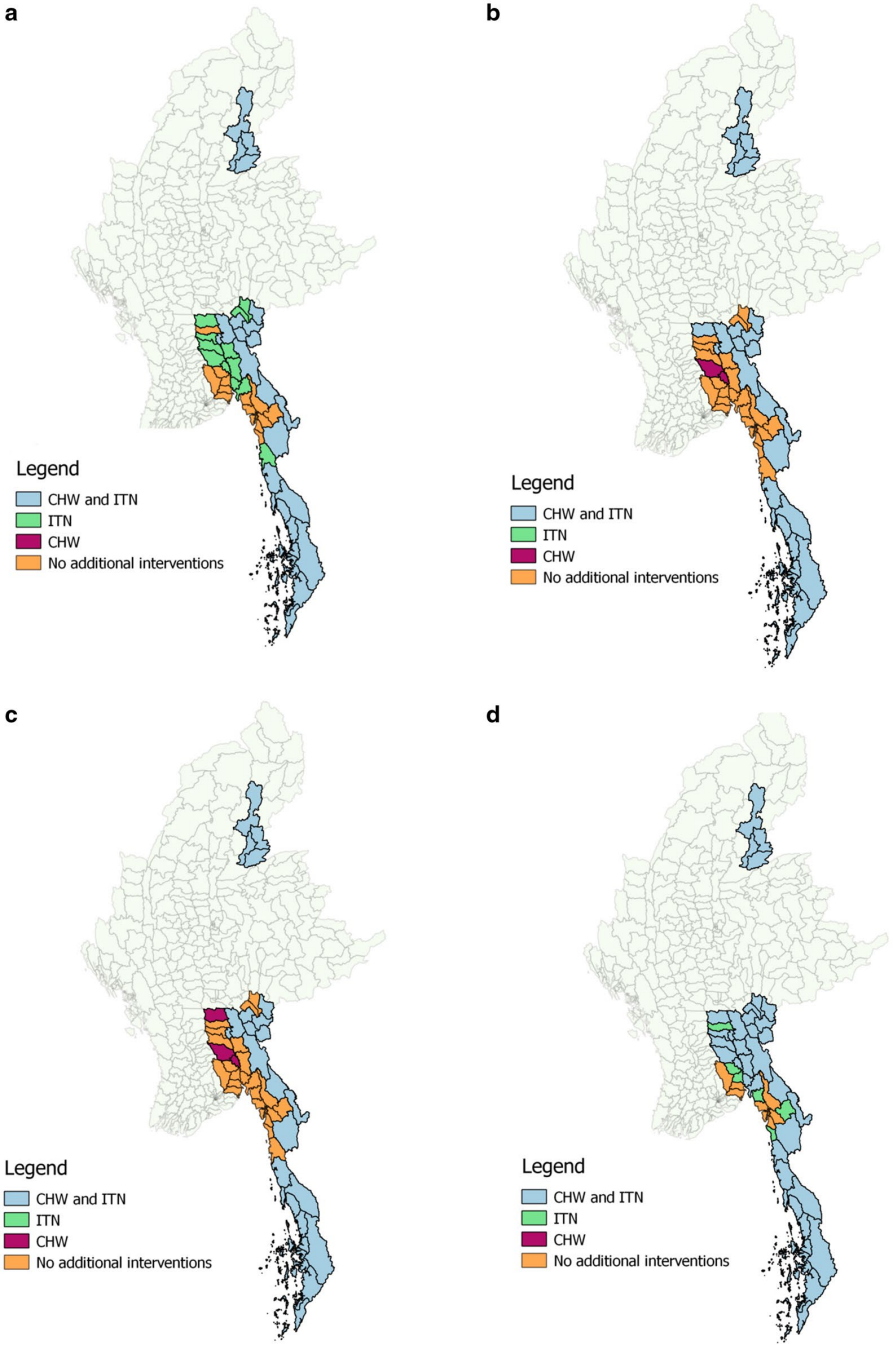
Township allocation of malaria interventions in the MARC region, Myanmar. Legends: Maps indicate allocation of US$ 10 million to bed nets and malaria community health workers in the MARC region, Myanmar. **a** Allocation using default parameter values detailed in Table 1. **b** Allocation assuming a lower bed net protective effect of 5 %. **c** Allocation assuming a higher uptake of community health workers; 95 % of malaria infections. **d** Allocation assuming 50 % cost-sharing for community health workers. For *panels* (**b**–**d)** all parameters other than the specified variation are the default values outlined in Table 1

Figure 5a presents an illustrative optimal allocation of an annual budget of US$ 10 million to CHW and ITN roll out in the 52 townships of the MARC region, Myanmar. Almost half of the townships are allocated both CHW and ITN, 12 townships receive ITN only and 15 townships are allocated to provide standard health services without CHW or ITN. Figure 5b–d present the scenario variations where key assumptions are varied in order to observe the effect on resource allocation. Panel b assumes a low ITN protective effect of 5 %, rather than the default 30 %. Panel c presents resource distribution assuming 95 % uptake of CHW by individuals with malaria, rather than 30 %. Panels b and c find that at the margin, CHW rather than ITN should be prioritized. The specific townships receiving these marginal interventions is likely to be an artefact of population size and the residual budget amount at the end of the allocation process. Panel d presents a cost-sharing scenario, where the benefits of an integrated CHW programme are represented by an assumption that funds allocated for malaria control need only fund 50 % of the total programme cost. Notably, the allocation of both CHW and ITN to the majority of Southern, and Western township and to the Kachin townships in the North, is robust to these scenario variations.

## Discussion

Malaria intervention decisions in Myanmar are based on judgement supported by the limited available evidence. The average and incremental cost effectiveness ratios give decision makers a sense of “bang for buck” to inform these judgements while the resource allocation modelling highlights the importance of targeting both interventions to where they can have the greatest impact. This study finds that CHW have the potential for high impact on malaria, particularly in difficult to access areas, where availability of other services may be low and if CHW use is good. However, CHW are more costly and, if only delivering malaria services, are associated with higher cost-effectiveness ratios. ITN are a robustly cost effective intervention but the total health impact is expected to be lower in Myanmar due to the biting habits of the of the main mosquito vector species. The annualization of the ITN cost over the lifespan of the net, conservatively assumed to be three years, means the comparative cost is lower. Although the cost of health gains is low with ITN, in the context of planning for malaria elimination more impactful interventions will need to be considered.

The cost effectiveness of both CHW and ITN is sensitive to the baseline availability of treatment, indicating that services will be most cost effective when targeted to areas with poor access to malaria diagnosis and treatment. The utilization of CHW is also very important and investment is quality training, CHW supervision and community engagement may be important to implementing a cost effective CHW programme [19]. A further option available to planners seeking to improve the cost effectiveness of CHW programmes is to expand the package of services offered by CHW. This is already happening and many CHW are now also providing a basic health care package or providing additional services such as tuberculosis detection and treatment. Measures to improve the cost effectiveness of community health workers include expanding the scope of available services; strategies to improve the likelihood that community members seek treatment from the community health worker when they have fever; and targeting community health workers to where they will be most cost effective.

For several reasons the main analysis does not apply a cost effectiveness threshold. It is difficult to define an appropriate threshold for the cost per YLL or DALY averted; the budget context in Myanmar is complex with modest NMCP funds being supplemented by international aid. Moreover in the context of a drive towards elimination all interventions will cease to appear cost effective as the malaria burden decreases (in absence of a model for long term benefits). The use of measures such as cost-per DALY averted are, therefore, less relevant and highly uncertain [20, 21]. The most immediately relevant question is how to maximize impact from malaria funds available in Myanmar and for this no threshold is necessary.

An extension of standard cost effectiveness analysis to spatially (in this case township-wise) specific resource allocation modelling highlights the need for a paradigm shift in policy discussion from prioritizing universal coverage of the “most cost effective” intervention to targeting of both interventions and presents illustrative township specific recommendations. In this analysis, malaria burden and to a lesser extent population numbers determine the optimal distribution of resources. Future work will seek to include additional data specific to each township.

Part of the aim of this study is to formalize through a cost effectiveness framework the kind of intuitive judgements that many policy makers and influencers in Myanmar are discussing. There has been much debate regarding the various merits of bed nets and malaria CHW. This paper does not come down on either side of this debate but seeks to summarize the characteristics of each and highlight the importance of targeting both to areas where impact can be maximized.

## Limitations

This study has several limitations. The model does not include human population movement or malaria transmission dynamics. A malaria transmission model, incorporated into the cost effectiveness model, would be a useful extension. This would allow indirect effects to be incorporated into the analysis and allow provide projections of the impact on malaria transmission going forward. The analysis does not include benefits to the patient beyond malaria impact, such as reduced costs to access care nor are issues of service quality examined here. For CHW there is a strong interest in extending their ability to diagnose and treat other causes of illness and therefore higher health gains than accounted for here. The model considers malaria control in the general population and does not specifically include high-risk groups such as migrant or mobile populations. Resource allocation modelling is applied at the township level whereas in Myanmar townships make decisions to allocate malaria interventions on a village-by-village basis. Finally, township variation here is characterized by population and malaria burden. Costs, baseline access to treatment and treatment-seeking behaviour are not assumed to vary between townships.

## Additional file

**Additional file 1.** Cost effectiveness and resource allocation of malaria control in Myanmar: further sensitivity and scenario analyses.
