# Supplementary material for: Cost effectiveness and resource allocation of Plasmodium falciparum malaria control in Myanmar: a modelling analysis of bed nets and community health workers
Source: Malar J. 2015 Sep 29;14:376. doi: 10.1186/s12936-015-0886-x (PMC4587798; doi:10.1186/s12936-015-0886-x)
Supplement: Supplementary file 1 — Additional file 1. Cost effectiveness and resource allocation of malaria control in Myanmar: further sensitivity and scenario analyses. [file 12936_2015_886_MOESM1_ESM.pdf]

Supporting material for the paper:

Cost effectiveness and resource allocation of malaria control in Myanmar:

A modelling analysis of bed nets and community health workers

Appendix 1: Univariate and Probabilistic Sensitivity Analysis

Three probabilistic analyses are included here, with important limitations. There is very little data available for more informative parametric sampling distributions. To err of the side of caution and methods are chosen to over-rather than under-estimate parameter uncertainty. Parameter values are sampled from uniform distributions between the ranges described in Table 1 in the main paper (n=1000). There is also no accounting for joint parameter variability; when two or more parameter values are not independent of one another. The probability of using a CHW may well be related to the availability of ACTs elsewhere, but at this time there is no data with which to characterise a joint distribution.

Figure S1 present a standard PSA analysis presented as a scatter plot on the cost effectiveness plane. Figure S2 presents the cost effectiveness acceptability curve (CEAC) using willingness-to-pay values between US$0 and US$3000 per DALY averted. Consistent colour coding is used throughout; Blue: bed nets; Green: community health workers and Orange: Both interventions.

Figure S1: Probabilistic sensitivity analysis of the cost effectiveness of malaria control and elimination in Myanmar

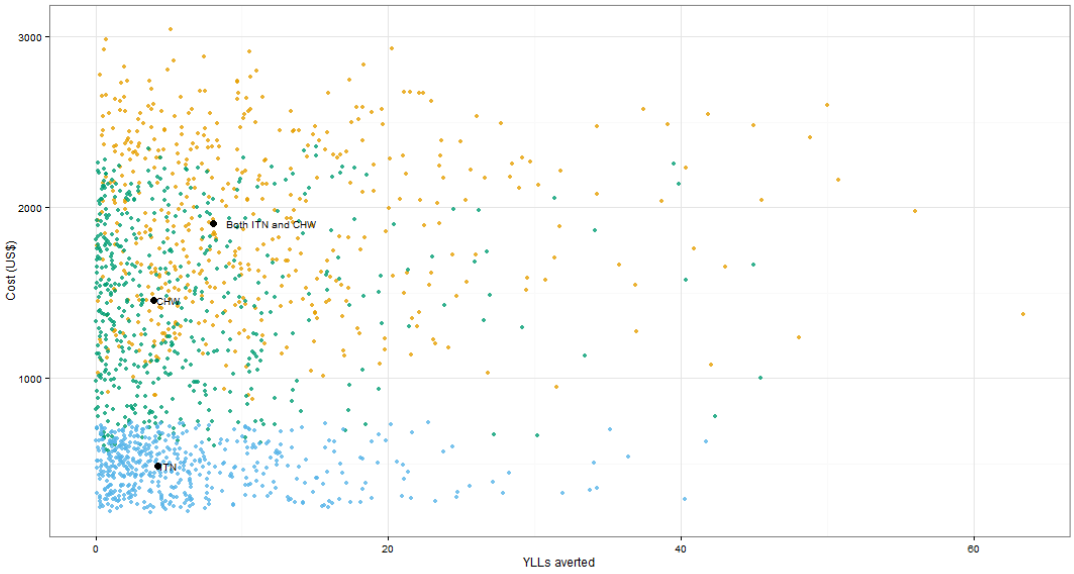

Figure S2: Cost effectiveness acceptability curve (CEAC) for malaria interventions in Myanmar

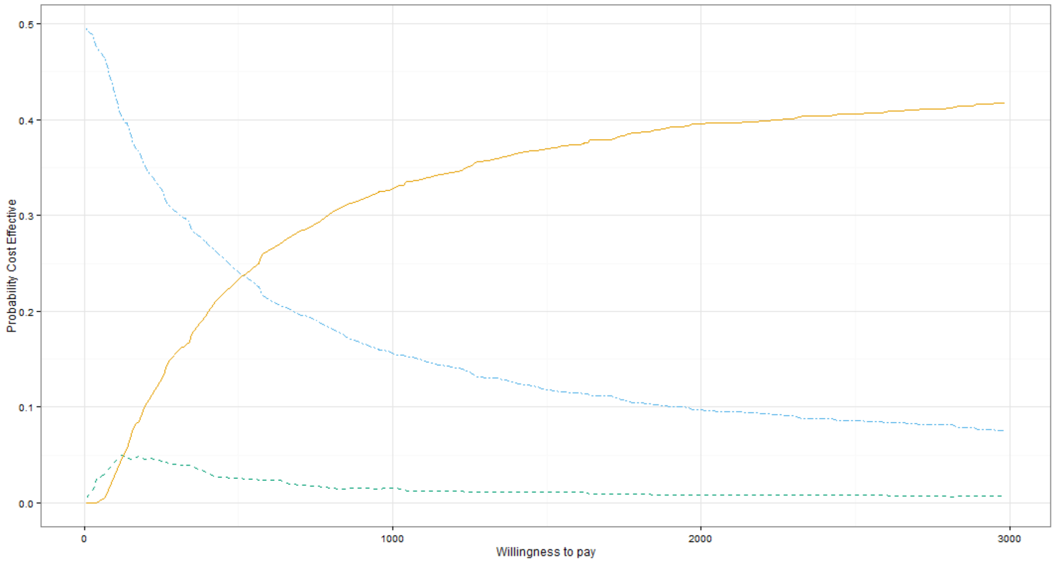

Secondly, a partial PSA was carried out within the bounds of the scenario analysis. In this case, the parameters defined by the scenario analysis can be considered considered 'variability' rather than uncertainty and as changes between settings are fixed for illustration of different scenarios. The probability of malaria is also considered to be a variable rather than uncertain parameter and is held constant at the default value. The PSA presents the residual uncertainty assuming the parameters defined by the scenario are known. It is worth noting that compared with the full PSA there is less overlap between intervention results, highlighting that better information key areas of variability, including remoteness, could improve decision making.

Figure S3: Scenario analysis and partial probabilistic sensitivity analysis of the cost effectiveness of malaria control and elimination in Myanmar

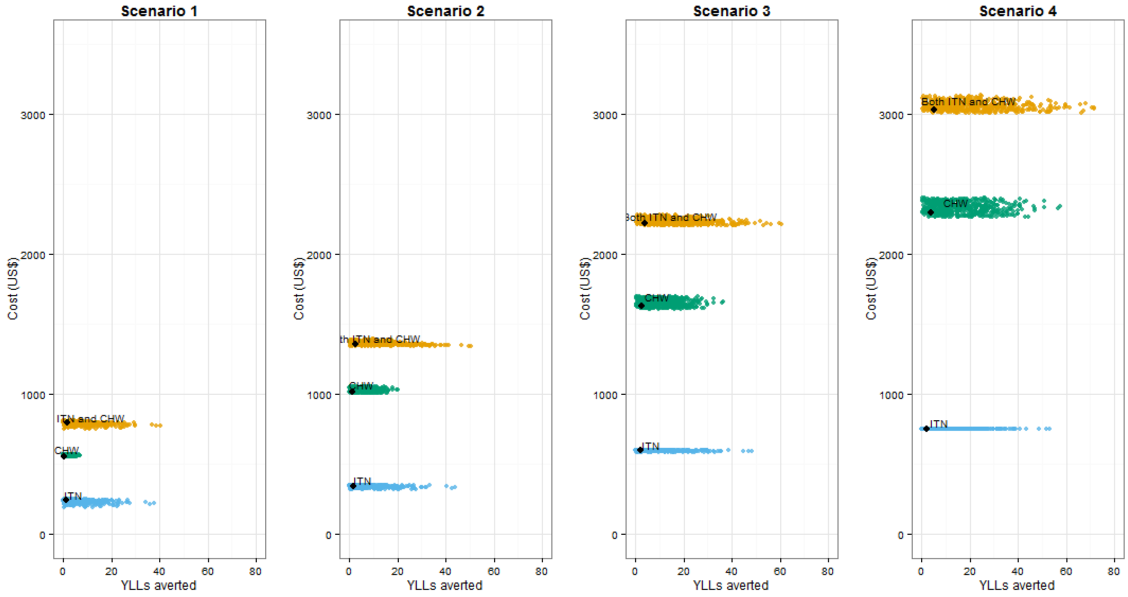

The above PSA analyses and provided for completeness and to conform to recommended practice in cost effectiveness analysis. However, as indicated in the main paper, the standard decision framework of choosing a single optimal intervention choice is not well suited to this context. Figure S4 presents a PSA as applied using the township resource allocation model. In this analysis the full resource allocation process is iterated 1000 times with parameter values except for malaria risk sampled randomly from the ranges specified in Table 1. Uncertainty in the township specific malaria risk was assumed to be due to a degree of under reporting. Malaria risk in each township is increased by between 1% and 10%, sampled at random from a uniform distribution separately for each township and repeated for each Monte Carlo simulation. This analysis is perhaps the most informative of the sensitivity analyses as it identifies some townships as clear priority investment areas, and conversely some townships that are unlikely to present value for money, under any combination of parameter values within the ranges specified in Table 1 in the main paper. All resource allocation simulations assume an annual budget of US$ 10 million.

Figure S4: Probabilistic uncertainty analysis of township allocation of malaria interventions in the MARC region, Myanmar

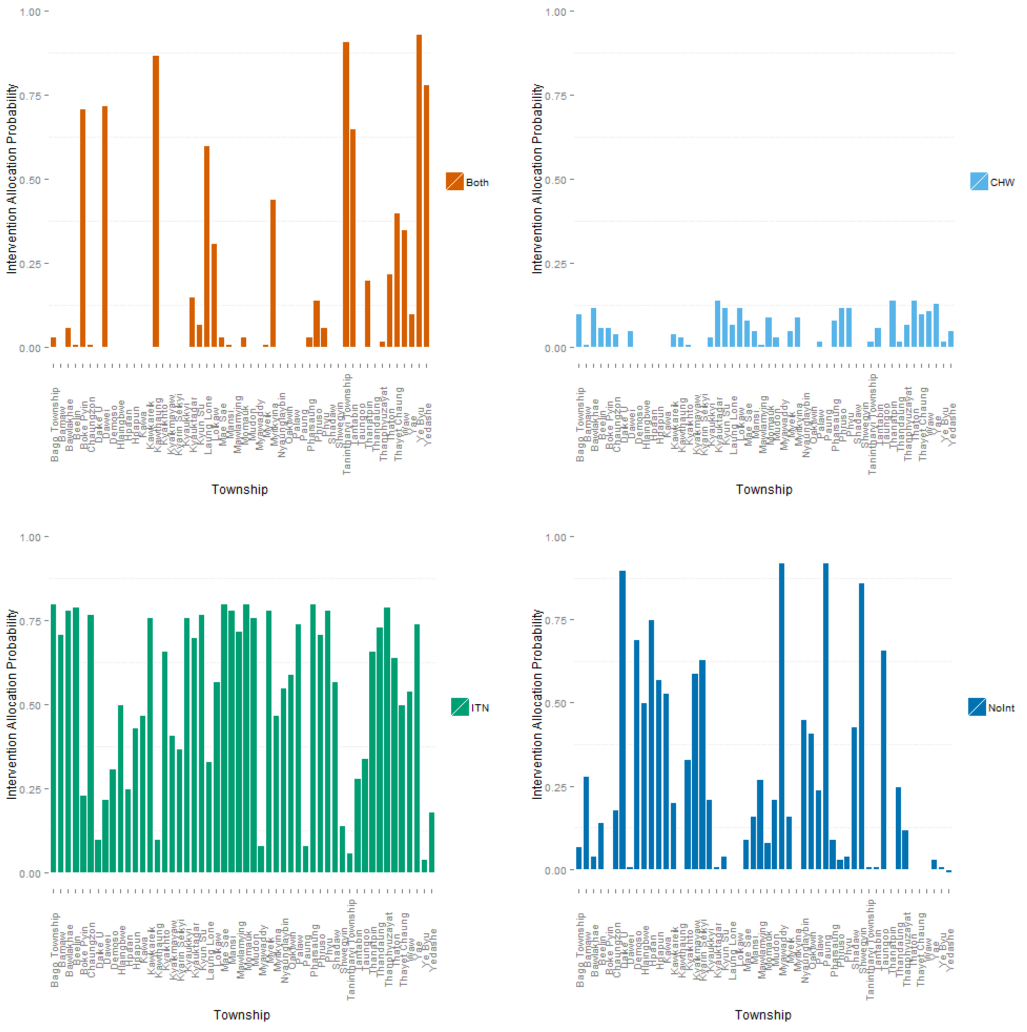

Appendix 2: Cost sharing for community health worker programmes

Some community health workers in Myanmar are trained to deliver not only malaria diagnosis and treatment but a range of additional health services. It is beyond the scope of this study to quantify the health impact of these services. Instead we present a cost sharing scenario, where one third of the cost burden is carried by malaria funding. Additional costs for further training or commodities are not included here. Here ITN are considered a malaria specific intervention and cost sharing is not applied.

Table S2 and Figure S6 show that with 1:2 cost sharing malaria to other services, CHW are now the most cost effective intervention in difficult and very difficult to access scenarios and also potentially cost effective in accessible and easily accessible scenarios. This illustrates the potential advantages of integrated service delivery and the resulting economies of scope. At the same time there is a limit to the capacity of CHW to take on additional responsibilities. Usually CHW also work full time to support themselves and their families.

Table S2: Costs and effects of malaria interventions in four remoteness scenarios with cost sharing for CHW

		**Remoteness: Easily Accessible**	**Accessible**	**Difficult to Access**	**Very Difficult to Access**
**ITN**	**Cost**	239	343	597	750
	**Effect**	135	171	198	225
	**CER**	1.77	2.01	3.01	3.33
	**ICER**	-	-	Dominated	-
**CHW**	**Cost**	183	335	537	757
	**Effect**	56	149	263	405
	**CER**	3.26	2.26	2.04	1.87
	**ICER**	0.70	0.36	-	0.04
**CHW & ITN**	**Cost**	422	678	1134	1507
	**Effect**	174	275	382	509
	**CER**	2.42	2.47	2.97	2.96
	**ICER**	4.66	3.22	5.01	7.25

*CER here compares costs and effects of an intervention compared with no intervention

** ICER compares costs and effects of an intervention compared with the next most effective undominated option

Figure S6: Costs and effects of malaria control in different accessibility scenarios with cost sharing for CHW

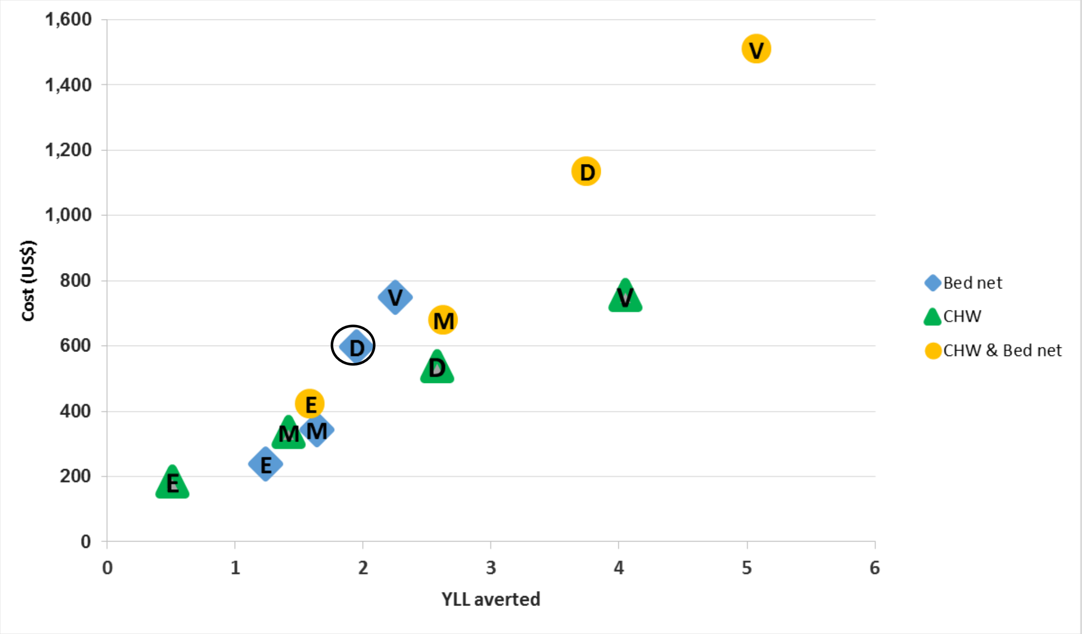

Appendix 3: Alternative model variant using "transmission days averted" as an effect metric

A limitation of the model used in the main body of this paper is the focus on direct impact. A more complex dynamic transmission model may be able to capture indirect effects. Here the model calculates effect size in the number of days of malaria transmission averted through treatment of cases or cases directly averted by bed nets. This metric was identified as a proxy for the impact of interventions on malaria transmission, rather than on direct health outcomes.

The probability tree (Figure S7) traces an individual through a chronological series of event possibilities beginning with an annual probability of contracting malaria (m) which is adjusted by the protective effect of ITN (p), if applicable. Individuals with malaria have a probability they will receive treatment from a provider other than a CHW (a). If a CHW is available in the village there is a probability (q) that a malaria case will seek treatment from the CHW, from both those who would have received treatment elsewhere and from those who would not have received any treatment. Each case of malaria has a mean number of malaria transmission days in the absence of treatment (t). We assume that non CHW treatment is with an ACT and that CHW treatment is with ACT plus primaquine. The reduction in transmission days is less for ACT treatment (r_2) than ACT plus primaquine (r_3) reflecting policy at the time of analysis. The terminal payoffs are scaled by village population (v) and calculate the net cost and net effects for each intervention arm for one village. Parameter values and ranges are found in Table S3.

Table S3: Parameter list and values for decision tree models

	Model parameter	Symbol	Default Value	Source
Setting	Baseline access to treatment (% of cases receiving ACT)	a	30%	2011 MARC survey indicates low ACT availability, but recently survey by PSI indicates a substantial increase.
	Cost of treatment	c_{ACT}	$3	Wholesale price of diagnosis and treatment, consumables only. 3MDG.
	Number of transmission days per case	t	30	Assumed a mean period of communicability of 30 days if untreated.
	Probability of getting malaria	m	5%	Probability of malaria is highly variable but changes do not affect comparative analysis between intervention options.
	Probability that a person with malaria uses a CHW (where available)	q	30%	Community survey by Department of Medical Research in Myanmar finds 19% of surveyed *first* seek treatment at CHW (unpublished). This parameter reflects the total treatment seeking proportion, not only the first place. Community survey in Cambodia finds low utilisation of CHW in villages with a CHW (Yeung *et al*. unpublished)
	Village population	v	500	Village size is based on unpublished unicef data. At the time of the study the village level census data was unavailable.
Intervention	Annual cost of ITN per person	c_{ITN}	$0.70	Estimated using financial reports from donors and implementing partners.
	Annual cost of CHW per person	c_{CHW}	$2	Estimated using financial reports from donors and implementing partners.
	ITN protective efficacy	p	30%	(5,7,15,16)
	Reduction in infectious days after treatment with ACT	r_2	80%	Assumed ACT treatment results is reduction from 30 to 6 transmission days.
	Reduction in infectious days after treatment with ACT+PQ	r_3	90%	Assumed ACT treatment results is reduction from 30 to 3 transmission days.

Figure S7 Transmission Days Averted Outcomes Decision Tree

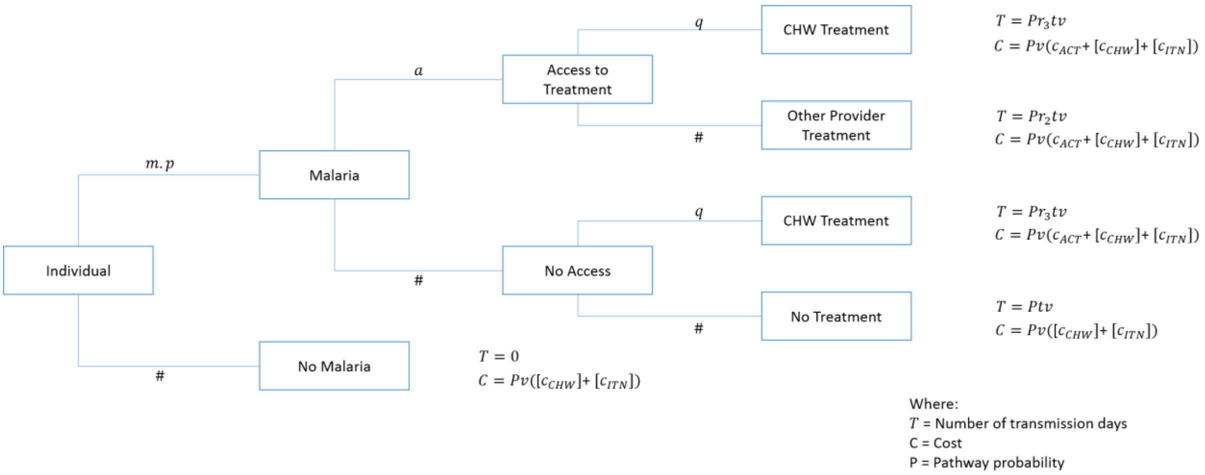

The trends in results are not substantially different from the model for DALYs averted. In the easily accessible village setting CHW avert 56 transmission days per year at a cost of US$ 556 (US$ 9.88 per transmission day averted). This rises in the very difficult to reach villages to 405 transmission days averted at a cost of US$ 2295 (US$ 5.67 per transmission day averted). Bed nets were consistently less costly and a modestly effective intervention. In the easily accessible village setting bed nets are predicted to avert 135 transmission days at a cost of US$ 239 (US$ 1.77 per transmission day averted), rising to 225 transmission days averted for US$ 750 (US$ 3.33 per transmission day averted). A combination of both bed nets and CHW in the very difficult to access village setting gives the greatest impact of 509 transmission days averted for a cost of US$ 3032 (US$ 5.96 per transmission day averted). The above results are summarised in Table S4 and Figure S8 and assume here that CHW only provide malaria services.

Table S4: Costs and effects of malaria interventions in four remoteness scenarios

		Remoteness: Easily Accessible	Accessible	Difficult to Access	Very Difficult to Access
**ITN**	**Cost**	239	343	597	750
	**Effect**	135	171	198	225
	**CER**	1.77	2.01	3.01	3.33
**CHW**	**Cost**	556	1016	1629	2295
	**Effect**	56	149	263	405
	**CER**	9.88	6.84	6.19	5.67
	**ICER**	Abs. Dominated	Abs. Dominated	Ext. Dominated	8.58
**CHW & ITN**	**Cost**	793	1354	2217	3032
	**Effect**	174	275	382	509
	**CER**	4.55	4.93	5.80	5.96
	**ICER**	14.07	9.73	8.79	7.12

*CER here compares costs and effects of an intervention compared with no intervention

** ICER compares costs and effects of an intervention compared with the next most effective undominated option

Figure S8: Costs and effects of malaria control in different accessibility scenarios

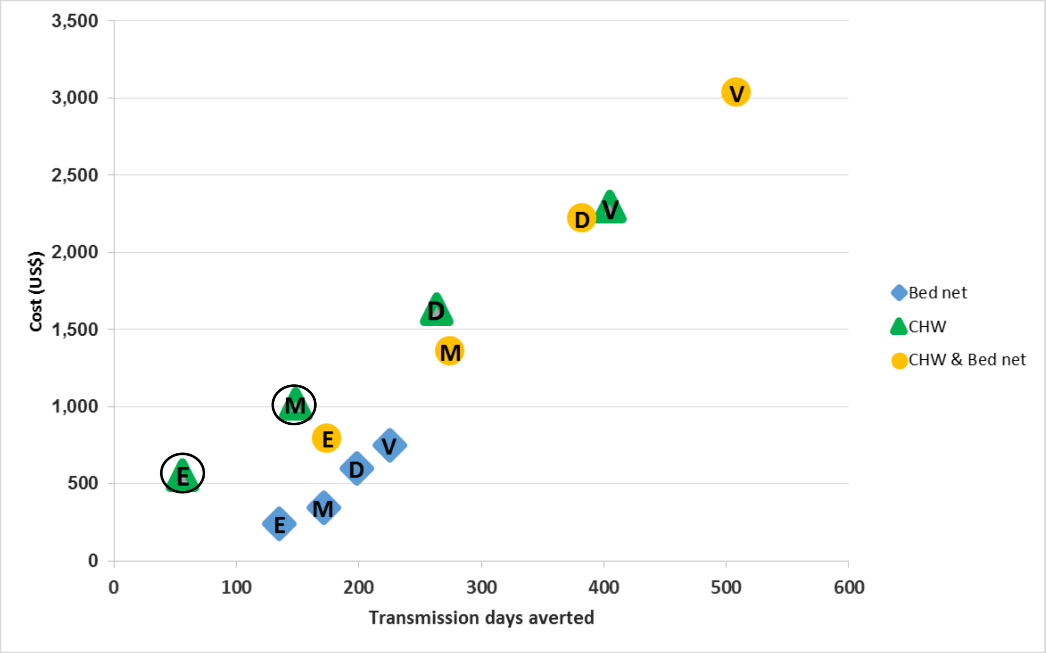

* Circle indicates a dominated intervention.
